# Dr. Adams on Tetanus

**Published:** 1830-10-01

**Authors:** 


### XVII.

### Dr. Adams on Tetanus.

In the Glasgow Medical Journal of last quarter, No. X. Dr. Adams, of Barrhead, has published a short, but sensible paper on tetanus, in which he has given tolerable specimens of the discrepant opinions and practices of medical men in this formidable disease. Without adducing any facts, or indeed opinions of his own, Dr. Adams has translated and condensed from a German author, (Dr. Michael Funk,) a few cases, with the reports of their dissections, leaving to the profession the option or task of drawing their own conclusions. The following are the abstracts of the cases.

"*Case* 1*st*. Francis Leuxner, 18 years of age, and of a vigorous constitution, was employed at a sheep-washing, 26th May, 1819. During the time he was employed, he felt the heat of the sun very strong upon his back. To save a lamb from drowning, he jumped into the water up to the neck. A few days afterwards, after a rigor and hot fit, pains were felt in his neck, and throughout the whole course of his back, and shortly after, the symptoms of trismus made their appearance. Opisthotonos appeared about the 7th or 8th day. He was bled to 30 ozs. had a purgative, and then some opium. The disease proceeded rapidly, without the smallest mitigation from the treatment, and on the morning of the 5th June, death took place amidst convulsions.

On the 6th, the body was opened. The arches of the vertebræ were removed from the first cervical to the last lumbar. In the cervical region, the dura mater was much reddened;—about the first dorsal vertebræ, and under, the canal was filled with extravasated blood, which had also extended some way along the nerves;—the dura mater very red. In the lumbar region this effusion was greatest, and here the dura mater was considerably thickened. When the dura mater was slit open, there flowed out about ℥iss. of dark bloody coloured serum. The spinal cord was now taken out with its coverings, and the dura mater slit up throughout its whole extent. The surface of the cord was rose red—the origins of the nerves swelled—and the cauda equina much reddened. The cord was harder than natural, and when cut into, was found much reddened. The substance of the sciatic nerve was much reddened, and exhibited a fine net-work of vessels.

*Case 2d.* Anna Ament, 19 years of age, of a strong constitution, received a wound in one of her fingers, from a splinter of wood. The wound inflamed severely, and an abscess formed, which was opened. A few days afterwards, she exposed herself while warm, to a current of cold air, at an open window. She had rigors, succeeded by a hot stage, pain in the neck, and spasms of the face: opium and carbonate of potash were administered. Next day the spasms and pains were increased, the mouth more closed, with one of its corners distorted, and the pain extended down the back. After a warm bath, the spasms and pain abated, and she could swallow better, but this relaxation was only of short duration. V.S. to ℥xxx. produced no change—the blood was very firm and buffy. In the evening she was again bled to ℥xx. and the finger was amputated. All the symptoms, however, became more severe, the patient expired on the 3d day.

'The distinguished Dr. Hesselbach inspected the body.' In some places, the nerve of the arm was remarkably inflamed. The whole vertebral canal was laid open;—between the dura mater and arachnoid, was contained a pretty large quantity of bloody serum, the vessels were very much injected, and some extravasation throughout the whole course of the spine. The spinal cord had a more than natural firmness, and was, throughout, from medulla oblongata to cauda equina, dyed red.

*Case 3d.* John Schäfer, aged 59, entered the hospital of Bamberg with typhus fever. Soon after his admission, he was seized with tetanus—severe pains in small of back—and paralysis of both upper and lower extremities: and he died in a state of opisthotonos on the 9th day after his admission. The fever had been very severe.

The dura mater of the spinal cord was much reddened—when slit up, 4 ozs. of bloody serum flowed out—a luxuriant net-work of injected vessels was seen upon the cord—the cauda equina highly reddened—and all the nerves of a pale red colour. The cord, when slit up, was reddish, and a good many bloody points appeared upon the cut surface—it was rather firmer than natural; but in all other respects, perfectly normal.

*Case 4th.* Eva Wolf, aged 36 years, was admitted into the hospital with typhus. The fever was severe, and with petechiæ. Tetanus seized her, which ended in paralysis, and the patient died on the 22d day, retaining complete consciousness.

The thoracic and abdominal viscera were found in a normal condition:—the dura mater of the cord tightly stretched, and its cavity filled with a great quantity of reddish serum, which flowed when slit up;—the pia mater was much reddened; the cauda equina was pale red;—and the substance of the cord rather firm, and upon being cut, it was found reddish.

*Case 5th.* John Seidlein, aged 36 years, received a wound from a horse's foot upon the left temple. Three days after, he had slight symptoms of trismus, and fever, with constipation, which were shortly followed by complete opisthotonos, and 7 days after receiving the wound he died.

He had been bled to 2lbs, and the blood had the buffy coat.

The thoracic and abdominal viscera were healthy. The dura mater of the cord appeared upon the stretch, and when divided, a small quantity of serum made its escape. Upon the cord, the vessels were observed very much injected, and even a little light-coloured extravasation upon it;—this inflammation extended to the medulla oblongata:—it was most considerable in the region of the neck, and less so in the cauda equina, although it was pretty much reddened. When cut through the cord showed considerable redness, and a good many bloody points upon the cut surface. In the brain, the vessels were observed considerably enlarged, though not to such a degree as one could pronounce it inflammation.

*Case 6th.* Sebastian Reh, aged 20 years, of a strong constitution, after having been heated with labour, exposed himself to cold. The following day he had rigors and pains in the neck,—on the 3d day, trismus. In a few days, the pain extended along the whole course of the spine, and he was seized with opisthotonos. He was treated antiphlogistically, and died 7 days after the attack of opisthotonos.

After death, an eruption was observed over the whole of the spine, resembling small blisters and phlyctenæ, but filled with fœtid air. The flesh was flabby, *and had a peculiar smell, like that of a bird of prey*—a smell which Dr. F. says he has remarked in his dissections of all similar cases. A considerable quantity of serum was found in the vertebral canal externally to the dura mater: in this situation was also found some bloody effusion, (coagulable lymph,) having the consistence of false membrane. Upon slitting open the dura mater, which was reddened, some serum was also found, and upon the cauda equina, some bloody effusion. Upon the cord was a fine network of vessels much injected. The cord was harder than natural. No morbid appearances were observed in the brain, or in the thoracic and abdominal viscera."

We agree with Dr. Adams that, if ever we are to know what is the disease with which we have to deal, it is by accumulation of cases and dissections, from which we are to draw cautious, just, and logical deductions.

---

